# Recent trends in the elucidation of complex triterpene biosynthetic pathways in horticultural trees

**DOI:** 10.1093/hr/uhae254

**Published:** 2024-10-08

**Authors:** Sandeep Dinday

**Affiliations:** Metabolic engineering and Synthetic Biology Laboratory, Department of Natural Products, National Institute of Pharmaceutical Education and Research, S.A.S Nagar 160062, Punjab, India

## Abstract

Triterpene (C30 isoprene compounds) represents the most structurally diverse class of natural products and has been extensively exploited in the food, medicine, and industrial sectors. Decades of research on medicinal triterpene biosynthetic pathways have revealed their roles in stress tolerance and shaping microbiota. However, the biological function and mechanism of triterpenes are not fully identified. Even this scientific window narrows down for horticultural trees. The lack of knowledge and a scalable production system limits the discovery of triterpene pathways. Recent synthetic biology research revealed several important biosynthetic pathways that define their roles and address many societal sustainability challenges. Here, I review the chemical diversity and biosynthetic enzymes involved in triterpene biosynthesis of horticultural trees. This review also outlines the integrated Design-Build-Test-Learn (DBTL) pipelines for the discovery, characterization, and optimization of triterpene biosynthetic pathways. Further, these DBTL components share many fundamental and technical difficulties, highlighting opportunities for interdisciplinary collaboration between researchers worldwide. This advancement opens up unprecedented opportunities for the bioengineering of triterpene compounds toward development and scaleup processes.

## Introduction

Triterpene with >20 000 structures represents a major group of natural products [1]. Plants produce numerous uncharacterized triterpenes that are often challenging to produce by synthetic chemistry. Engineering plants for sustainable bioproduction of triterpenes has become of great interest and requires attention [1, 2]. Triterpenes are majorly found in three forms: oxygenated, glycosylated, and acylated ones [3], and exhibit diverse biological activities such as anti-diabetic, anti-inflammatory, and anti-cancer [4]. These complex forms are synthesized by modifying the triterpene scaffolds with regio-specific and stereo-selective decorations such as oxidation, glycosylation, and acylation [5–7]. However, the natural reservoir of triterpenes remains mostly unexploited and requires an understanding of the target enzymes.

The triterpenes are biosynthesized by joining six C-5 isoprene units to form a C-30 linear compound, squalene. In prokaryotes, squalene acts as a direct precursor for hopanoid biosynthesis. However, in eukaryotes, the squalene is initially activated into 2,3-oxidosqualene, which is further cyclized through a group of enzymes known as oxidosqualene cyclases (OSCs) to form diverse triterpene scaffolds [8–10]. These triterpene scaffolds are utilized by other groups of enzymes such as cytochrome P450 monooxygenases (P450s)/2-oxoglutarate-dependent dioxygenases (2-ODDs) for oxygenation, UDP-glycosyltransferases (UGTs) for glycosylation and acyltransferases (ATs) for acylation [1, 4, 6]. These chemical decorations generate unique triterpenoid structures with enhanced bioactivities [11–13]. Due to this huge diversity in triterpene structures, it is not surprising that these compounds have been used in different economic sectors ranging from medicine to agriculture and industries [14, 15].

**Figure 1:**
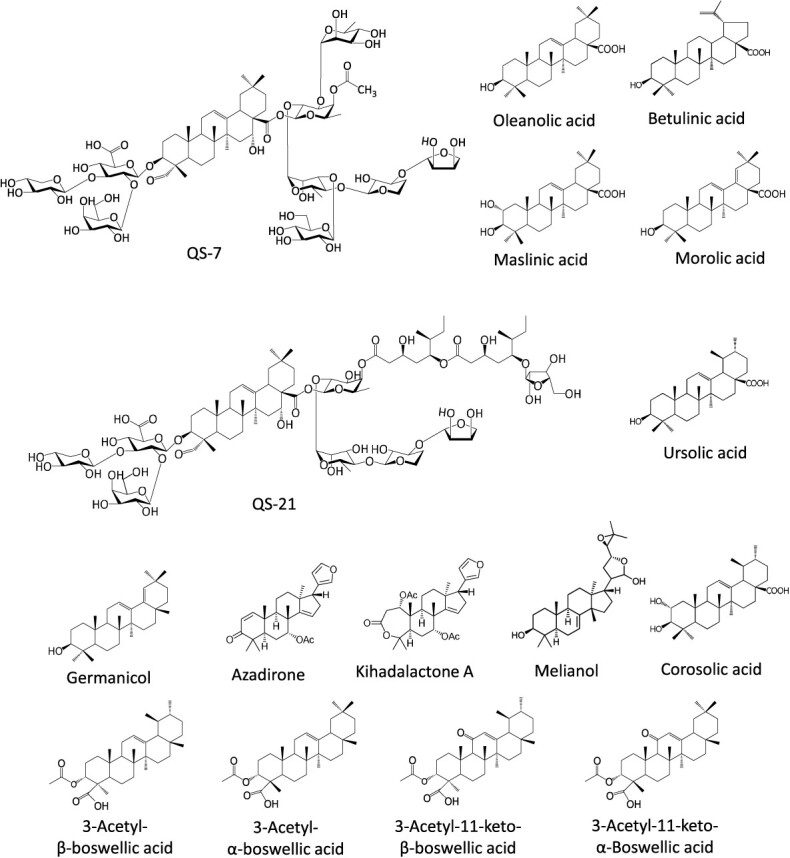
**Representative structure of triterpene compounds isolated from horticultural trees.** Multi-omics approaches were utilized for the identification and functional characterization of triterpene biosynthesis genes, either leading to complete pathway elucidation or any specific enzymatic step of the pathway.

Considering numerous complex triterpene structures in plants; it is quite possible that many biosynthetic pathway enzymes are yet to be identified. Generally, triterpenes are harvested using traditional methods from native hosts or produced by organic synthesis. The triterpene structural complexity with multiple stereocenters and functional moieties makes their chemical synthesis unattractive. These methods are inefficient at the industrial level and often lead to high cost and low quantity. The concept of single-compound medicine is responsible for developing synthetic biology platforms and underpins the modern pharmaceutical paradigm. Synthetic biology along with advanced analytics, computational technologies, and molecular biology approaches have shown that triterpenes are formed through complex metabolic grids directed by promiscuous pathway enzymes. The production of triterpene compounds in heterologous hosts, such as yeast and *Nicotiana benthamiana*, is emerging as an attractive alternative. Triterpenes have gained considerable attention from the scientific community due to their therapeutic importance, and significant progress has been made toward the discovery of biosynthetic genes and enzymes. Still, only a few triterpene pathways such as azadirone, betulinic acid, corosolic acid, kihadalactone A, maslinic acid, melianol, morolic acid, oleanolic acid, ursolic acid, QS-7, and QS-21 are discovered from horticultural trees (Fig. 1). The purpose of this review is to highlight recent developments in the area of triterpene biosynthesis and clarify the role of structural decoration enzymes in the biosynthetic pathway. This review provides a crucial foundation for the exploration of undiscovered triterpenes in horticultural trees and also discusses the rationale of the integrated DBTL approaches leading to the biotechnological production of triterpenes.

## Triterpene biosynthetic pathway

### Early pathways to the scaffolds

**Figure 2:**
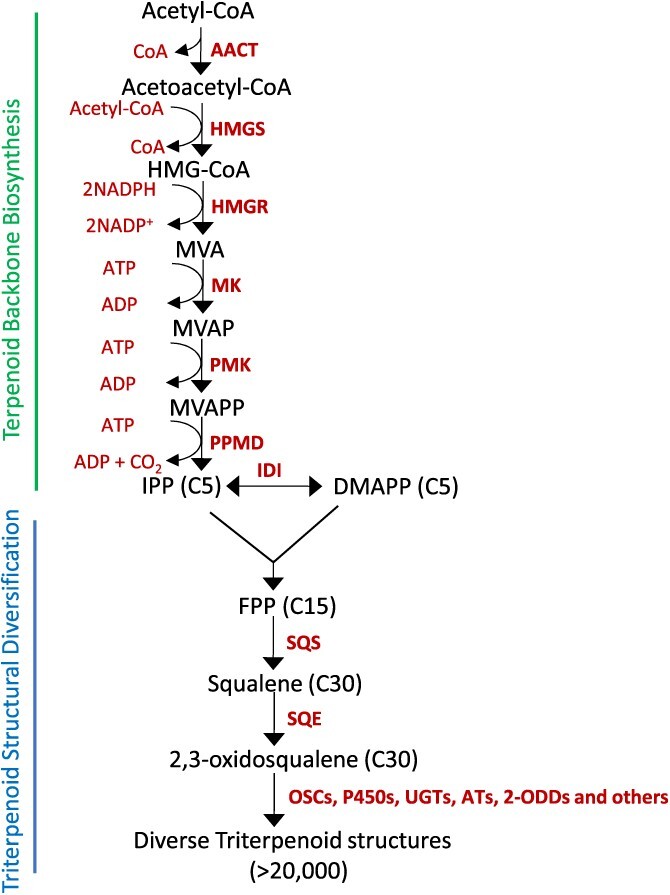
**Triterpene biosynthetic pathway in plants.** The MVA pathway generates 5-carbon terpenoid precursors, IPP, and DMAPP, which are converted to 2,3-oxidosqualene. In the late triterpenoid pathway, the triterpene structures are diversified with the sequential action of OSCs, P450s, UGTs, ATs, 2-ODDs, and other scaffold-decorating enzymes. Abbreviations: AACT, acetoacetyl-CoA thiolase; HMGS, 3-hydroxy-3-methylglutaryl-CoA synthase; HMG-CoA, 3-hydroxy-3-methylglutaryl-CoA; HMGR, 3-hydroxy-3-methyl- glutaryl-CoA reductase; MVA, mevalonate; MK, mevalonate kinase; MVAP, mevalonate 5-phosphate; PMK, phosphomevalonate kinase; MVAPP, mevalonate 5-diphosphate; PPMD, diphospho-mevalonate decarboxylase; IPP, isopentenyl diphosphate; IDI, isopentenyl diphosphate isomerase; DMAPP, dimethylallyl diphosphate; FPP, farnesyl diphosphate; SQS, squalene synthase; SQE, squalene epoxidase; OSCs, oxidosqualene cyclases; P450s, cytochrome P450 monooxygenases; UGTs, UDP-glycosyltransferases; ATs, acyltransferases; 2-ODDs, 2-oxoglutarate-dependent dioxygenases.

Several excellent reviews cover terpene biosynthesis in detail, so here the topic is discussed briefly [1, 4, 6, 16, 17]. Plants contain a vast array of terpene with complex structures and biosynthetic pathways [17]. Generally, terpene skeletons are formed from the cytosolic mevalonic acid (MVA) pathway and plastidial Methylerythritol 4-phosphate/deoxyxylulose-5-phosphate (MEP) pathway [18, 19]. MVA pathway is majorly involved in triterpene backbone synthesis and initiates with the acetyl Co-A and gradually forms isopentenyl pyrophosphate (IPP) (Fig. 2). However, the MEP pathway produces gibberellins, diterpenoids, carotenoids, and chlorophylls. The multistep MVA pathway catalyzes the conversion of acetyl Co-A to acetoacetyl-CoA by the enzyme acetoacetyl-CoA thiolase (AACT). Further, acetoacetyl-CoA and acetyl-CoA are condensed to generate one molecule of 3-hydroxy-3-methylglutaryl-CoA (HMG-CoA). This reaction is mediated by the enzyme HMG-CoA synthase (HMGS). After that, HMG-CoA reductase (HMGR) causes reductive diacylation of HMG-CoA to form MVA. Further, two repetitive rounds of phosphorylation of MVA generate mevalonate-5-diphosphate (MVAPP) by mevalonate kinase (MK) and phosphomevalonate kinase (PMK). Finally, MVAPP undergoes ATP-dependent decarboxylation to form IPP by mevalonate diphosphate decarboxylase (PPMD) [4, 20].

### Triterpene structural diversification

The triterpene backbone is biosynthesized from IPP and its allylic isomer dimethylallyl pyrophosphate (DMAPP). IPP (two molecules) and DMAPP (one molecule) are condensed to form farnesyl pyrophosphate (FPP). Finally, squalene synthase (SQS) condenses the two molecules of FPP (15-C) to form squalene (30-C), which further undergoes epoxidation to form 2,3-oxidosqualene by squalene epoxidase (SQE). 2,3-Oxidosqualene acts as a mutual precursor for triterpenes and phytosterols biosynthesis, and is cyclized to form a variety of polycyclic skeletons by OSCs. Due to this reason the step catalyzed by OSCs is marked as the branch point between primary (phytosterols) and secondary metabolism (triterpenoids). The enzyme cycloartenol synthase (CAS) is responsible for the cyclization of 2,3-oxidosqualene to cycloartenol (primary triterpene precursor). Further, cycloartenol generates a mixture of phytosterols including cholesterol, campesterol, and sitosterol. The steroidal saponins utilize cholesterol to form spirostanol or furostanol derivatives. The nature of triterpene biosynthetic enzymes lends itself naturally to the complex metabolic networks. Even a single or few changes in the amino acid sequences of an OSC can alter the product profiles [21]. OSCs can produce dozens of cyclized products [22, 23]. Further, site-directed mutagenesis increases the product specificity and catalytic activity. After OSC-medicated cyclization, the triterpene backbones are often decorated by P450s, UGTs, ATs, and 2-ODDs, and add bioactive moieties [1, 4, 5, 24, 25]. The addition of functional moieties in triterpene structures increases the polarity and bioactive properties. Specifically, P450s are known to oxygenate the substrates in multiple positions and related triterpene backbones. Therefore, P450s are recognized as the gatekeepers in the pathway bifurcation of industrially important triterpenes [26]. P450s provide anchor points for UGTs, ATs, and 2ODDs to produce >20 000 triterpene structures. This functional promiscuity of enzymes with related intermediates is the key to creating a complex triterpene network in plants [27]. Thus, it is quite evident that a single plant species can produce many triterpenes. Although, several groups of enzymes have been discovered for their roles in triterpene biosynthesis. Still, only a few have been identified from horticultural trees and there is a great scope to work in this direction.

### Select triterpene biosynthetic pathways in horticultural trees

Traditionally, triterpenes are acquired through plant extraction and chemical synthesis. However, these strategies are time- and energy-consuming. Recently, the synthesis of triterpenoids has been achieved in heterologous hosts including prokaryotic (*Escherichia coli*) and eukaryotic (*Saccharomyces cerevisiae* and *N. benthamiana*) [28, 29] (Table 1). These heterologous hosts are known for their robustness, precursor supply, and genetically tractable system. Overall, the reconstitution of triterpenoid biosynthesis genes in heterologous hosts stands out as an ideal system and requires optimization for commercial applications. At present omics studies have been implemented in horticultural trees for medicinal and fruit use, but there is great scope to extend this to other categories of horticultural trees valued for ornamental, landscape, and nut production. Therefore, this section discusses the discovered triterpene biosynthetic pathways in horticultural trees.

**Table 1:** Available resources for triterpene in horticultural trees.

**Target plant**	**Target compound**	**Target genes**	**Strategies used**	**Heterologous hosts**	**References**
*B. forficata*	α-Amyrin, β-Amyrin, Germanicol and Lupeol	OSCs	Transcriptomics and homology-based cloning	*S. cerevisiae* and *N*. *benthamiana*	[30]
*B. serrata*	3-Acetyl-β-boswellic acid, 3-Acetyl-α-boswellic acid, 3-Acetyl-11-keto-β-boswellic acid and 3-Acetyl-11-keto-α-Boswellic acid	AT	Transcriptomics, metabolomics, and gene function analysis	*E. coli* and *N*. *benthamiana*	[31]
*C. acuminata*	Betulinic acid, oleanolic acid and ursolic acid	P450s	Multiomics integrative analysis	*N*. *benthamiana*	[32]
*L. speciosa*	α-Amyrin, β-Amyrin, betulinic acid, corosolic acid, lupeol, maslinic acid, oleanolic acid, and ursolic acid	OSCs and P450s	Transcriptomics, metabolomics, and gene function analysis	*S. cerevisiae* and *N*. *benthamiana*	[33]
*M. domestica*	α-Amyrin, β-Amyrin, betulinic acid, germanicol, lupeol, morolic acid, oleanolic acid, and ursolic acid	OSCs and P450	Homology-based approach	*N*. *benthamiana*	[34]
*O. europaea*	α-Amyrin, β-Amyrin, corosolic acid, maslinic acid, oleanolic acid, and ursolic acid	OSCs and P450s	Genome mining, biochemical analysis, and trait-association studies	*N*. *benthamiana*	[35]
*Q. saponaria*	QS-7 and QS-21	OSCs, P450s, UGTs and ATs	Genome mining, co-expression of pathway genes, and biochemical analysis	*S. cerevisiae* and *N*. *benthamiana*	[36–38]
*T. arjuna*	α-Amyrin, β-Amyrin, and lupeol	OSCs	Transcriptomics, metabolomics, and gene function analysis	*S. cerevisiae*	[39]
*A. indica*, *C. sinensis*, and *M. azedarach*	Limonoids	OSCs, P450s, MOIs, ATs, SDRs, AKRs, and LFs	Genome and transcriptome mining, co-expression of pathway genes, and biochemical analysis	*N*. *benthamiana*	[40, 41]

#### Avicennia marina

**Figure 3:**
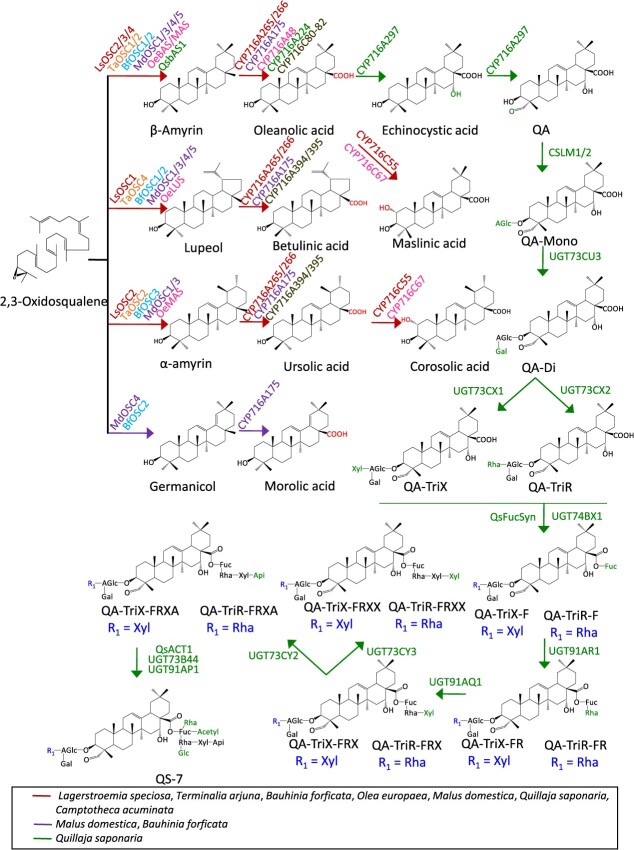
**Reconstitution of triterpene biosynthetic pathways of select horticultural trees.** The common triterpene pathway in *L. speciosa*, *T. arjuna*, *B. forficata*, *O. europaea*, *M. domestica*, and *Q. saponaria* are shown in red; the violet represents *M. domestica* and *B. forficata* and green represent *Q. saponaria*. Abbreviations: QA, quillaic acid; QA-mono, 3-O-{β-D-glucopyranosiduronic acid}-QA; QA-di, 3-O-{β-D-galactopyranosyl-(1 → 2)-β-D-glucopyranosiduronicacid}-QA; QA-TriX, 3-O-{β-D-xylopyranosyl-(1 → 3)-[β-D-galactopyranosyl-(1 → 2)]-β-D-glucopyranosiduronicacid}-QA and QA-TriR, 3-O-{α-L-rhamnopyranosyl-(1 → 3)-[β-D-galactopyranosyl-(1 → 2)]-β-D-glucopyranosiduronic acid}-QA.

Grey mangrove (*A. marina*) tree is utilized in traditional medicine systems for the cure of various diseases such as diabetes, cancer, rheumatism, smallpox, and ulcers [42, 43]. These bioactivities are due to the presence of pharmacological compounds including flavonoids, phenylpropanoids, steroids, iridoids, and terpenoids [43, 44]. However, the specific compounds responsible for different bioactivities remain unknown. In a recent study, transcriptome and triterpene profiling resulted in the identification and functional characterization of CYP716s, responsible for the decoration of amyrins (α/β-amyrin) and lupeol scaffolds [45]. CYP716A259 catalyzed the three-step C-28 oxidation reaction of α/β-amyrin and lupeol to form ursolic acid/oleanolic acid and betulinic acid. The other P450, CYP716C53, is characterized as C-2α hydroxylase that converts ursolic acid and oleanolic acid to corosolic acid and maslinic acid (Fig. 3). Overall, this study provides insight into triterpene biosynthesis in grey mangrove leaves.

#### Bauhinia forficata

*Bauhinia* is the largest genus in the Cercidoideae subfamily and comprises ~600 species, broadly distributed in most tropical countries [46]. *Bauhinia forticata* (commonly known as the Brazilian orchid tree) is native to South America and known for its diverse biological activities [47, 48]. Preclinical and clinical studies have suggested that the standardized leaf extract (yielding 2% total flavonoids per capsule) of *B. forficata* is useful as an adjuvant treatment in type-2 diabetes patients [49]. The tea prepared from *B. forficata* leaves (0.4% in 200 ml of water) used for 3 months in type-2 diabetes patients (mean age 62 years) decreases the levels of triglycerides and total cholesterol levels to 48 and 17 mg/dl [50]. The reduction in triglycerides and total cholesterol levels by 26% and 9% from baseline suggests its role as a complementary therapy in type-2 diabetes. To date, few studies have been done to investigate the beneficial triterpenes in *B. forficata*. In a recent study, transcriptome and heterologous expression studies were used to discover four OSCs, responsible for forming diverse triterpene profiles in *B. forficata* [30]. The four OSCs (BfOSC1–4) utilized 2,3-oxidosqualene, to produce β-amyrin, germanicol, α-amyrin, and cycloartenol (Fig. 3). BfOSC3 accounts for >95% of the total triterpenoids in *B. forficata* and is responsible for preponderant α-amyrin activity. Further, meticulous sequence analysis of OSCs revealed the presence of a TLCYCR motif in the *BfOSC3* gene. Replacing the TLCYCR motif with the MWCYCR motif through PCR-based site-directed mutagenesis studies alters the functionality of *BfOSC3* such that it no longer produces α-amyrin. Although the catalytic mechanism is not found in this study, these results facilitate the engineering of OSCs for producing the altered cyclized product. Collectively, these results provide novel insights into OSC-mediated cyclization in *B. forficata* and enable efficient OSCs for commercial production.

Further, the same research group utilize BfOSC2 (germanicol synthase) and coexpress it with *BvCYP716A49* (C-28 oxidase) from *Beta vulgaris* to form morolic acid in yeast [51]. Morolic acid is well known for its pharmacological activities and there is a great demand for commercial production. Therefore, a chimeric transcriptional activator (fusion of Gal4dbd.ER.VP16) was introduced to reconstruct the cellular galactose network. Further, triterpene precursors in the MVA pathway have been increased by overexpressing the truncated HMG1 (hydroxymethylglutaryl-CoA reductase) and upc2–1 (sterol-regulating transcription factor). As per the literature, this is the highest titer of morolic acid (20.7 ± 1.8 mg/l) produced in the yeast system. Utilizing a combinatorial approach to obtain these natural triterpenes is more promising and requires attention.

#### Camptotheca acuminata

*Camptotheca acuminata* (Chinese name that translates to ‘tree of joy’, ‘happy tree’, and ‘Xi Shu’) is well known for the discovery of the topoisomerase I inhibitor camptothecin [52, 53]. More than 100 metabolites have been isolated from the different parts of the *C. acuminata* tree. These metabolites belong to the alkaloids, flavonoids, and terpenoids classes of natural products [54]. About 26 triterpenes are known to accumulate in *C. acuminata*, including lupane-, oleanane-, and ursane-type. The lupane-, oleanane-, and ursane-type triterpenes are biosynthesized from pentacyclic precursors lupeol, β-amyrin, and α-amyrin. The presence of C-28 carboxy products such as betulinic acid, oleanolic acid, and ursolic acid in the plant indicates the existence of functionally active C-28 oxidases. Using the multi-omic database, five CYP716s (CYP716A394/395 and CYP716C80–82) were identified and functionally characterized [32]. *In vitro* activities using CYP716 microsomal proteins revealed that the CYP716C subfamily is responsible for oleanane (β-amyrin oxidation) biosynthesis. However, CYP716A showed ursane (α-amyrin) and lupane (lupeol) oxidation activity (Fig. 3). Further activities of CYP716 genes were verified in *N*. *benthamiana*, and the results agreed with the *in vitro* data. Overall, this study provides insight into CYP716s role in decorating the pentacyclic triterpenes structures in *C. acuminata*.

#### Lagerstroemia speciosa

*Lagerstroemia speciosa* (L.) Pers. (Jarul or Banaba, also known as Pride of India) can accumulate pentacyclic triterpenes in leaves. Leaf pentacyclic triterpenes, including corosolic, ursolic, betulinic, maslinic, and oleanolic acids, displayed various pharmacological activities [55–58]. Recently, a multi-omics approach including transcriptomic and metabolomic along with pathway reconstruction analysis has been used to identify OSCs and P450s involved in pentacyclic triterpene biosynthesis in banaba [33]. In this study, the red-colored leaves with higher amounts of ursane and oleanane triterpenes were used as optimum leaf material for RNA-guide gene discovery. Further, rigorous computational analysis led to the identification of functionally active five OSCs and three CYP716s involved in triterpene biosynthesis. Combinatorial analysis of OSCs and CYP716s in yeast and *N*. *benthamiana* revealed four monofunctional OSCs (LsOSC1,3–5) having product specificities for either lupeol/β-amyrin/cycloartenol and a multifunctional OSC (LsOSC2) responsible for preferential accumulation of α-amyrin. Two CYP716s (CYP716A265/266) were identified as α-amyrin/β-amyrin/lupeol C-28 oxidases and a CYP716C55 catalyzed C-2α hydroxylation step that converts ursolic acid and oleanolic acid to corosolic acid and maslinic acid (Fig. 3). Besides, seasonal variation is also studied by transcript and metabolite analysis, suggesting a major role of LsOSC2, CYP716A265, and CYP716C55 in determining ursane and oleanane levels in banaba leaves.

#### Malus domestica

Apple (*Malus* × *domestica*) accumulates bioactive pentacyclic triterpenes that belong to the ursane, oleanane, and lupane class [59]. In apple (‘Royal Gala’), a full-length expressed sequence tag database is used for the identification of three OSCs (MdOSC1–3) involved in the triterpene biosynthesis [60]. Among these two EST sequences, MdOSC1 and MdOSC3 were essentially identical having >99% amino acid similarity. The expression of *MdOSC1* and *MdOSC2* in yeast (*Pichia methanolica*) and *N*. *benthamiana* resulted in the accumulation of α-amyrin and β-amyrin (5:1) in MdOSC1. However, no product was identified in MdOSC2, suggesting that it is either encoded by pseudogenes or expression error. The presence of oleanane and lupane in apples suggested the presence of more OSC genes in the apple genome. Therefore, a homology-based approach was used for the identification and functional characterization of two new multifunctional OSCs (MdOSC4 and MdOSC5) and a P450 (CYP716A175) involved in triterpene biosynthesis [34]. In this study, MdOSC1,3–5 were expressed transiently in *N*. *benthamiana* to check the functionality of genes. The expression of previously identified MdOSC1 confirmed its multi-functional role. However, a small amount of lupeol is also detected, suggesting a new α-amyrin, β-amyrin, and lupeol (85:13:2) ratio. MdOSC4 cyclizes 2,3-oxidosqualene to germanicol, β-amyrin, and lupeol at a ratio of 82:14:4 and MdOSC5 forms lupeol and β-amyrin in proportion 95:5. Further, CYP716A175 acts as C-28 oxidase and utilizes lupeol, β-amyrin, α-amyrin, and germanicol to produce betulinic acid, oleanolic acid, ursolic acid, and putatively morolic acid (Fig. 3). Collectively, these studies suggested the key role of MdOSC1 and MdOSC5 in triterpene biosynthesis of apple fruit.

#### Olea europaea

Olive (*O. europaea*) fruits and oil bioactivities are largely attributed to triterpene such as oleanolic, maslinic, and ursolic acid. The cyclization of 2,3-oxidosqualene for olive triterpenes is carried out by a multifunctional OSC (OeMAS) having a product preference for α-amyrin [61] and two monofunctional OSCs (OeLUS and OeBAS) having product specificity for lupeol and β-amyrin [35, 62]. Recent studies on olive P450s reveal that CYP716A48 catalyzed C-28 oxidation of α-amyrin/β-amyrin/lupeol to produce ursolic acid/oleanolic acid/betulinic acid. CYP716C67 is responsible for C-2α hydroxylation of the oleanolic and ursolic acid to form maslinic acid and corosolic acid [35, 63] (Fig. 3). Combined approaches such as genome mining, homology-based search, biochemical analysis, and trait association studies have been used to identify triterpene pathway genes.

#### Terminalia arjuna

The Arjuna (*T*. *arjuna*) tree belonging to the Combretaceae family has been used in Indian traditional medicine for its health benefits. The tree accumulates bioactive triterpenes and saponins in a tissue-specific manner. Arjuna triterpenes are classified as lupane, oleanane, and ursane [64–66]. Oleananes extracted from bark extracts were considered major triterpenes and were suggested to have cardioprotective effects [66, 67]. Recently, a biochemical approach along with metabolomics and transcriptomics has been used to define a suite of four OSCs (TaOSC1–4) [39]. Among them, TaOSC1, TaOSC3, and TaOSC4 have biochemically converted the substrate, 2,3-oxidosqualene to β-amyrin, cycloartenol, and lupeol (Fig. 3). However, TaOSC2 was biochemically characterized as mixed amyrin synthase producing both α-amyrin and β-amyrin, having a product preference for α-amyrin. Further, the transcript analysis revealed that TaOSC1 transcript is preferentially expressed in bark, suggesting its role in oleanane biosynthesis in bark.

#### Quillaja saponaria

**Figure 4:**
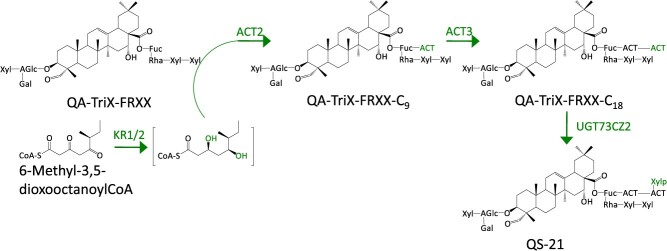
**Reconstitution of QS-21 biosynthetic pathway.** Genes from *Q*. *saponaria* are shown in green. Solid arrows denote single enzymatic steps, respectively. Abbreviations: AT (ACT), acyltransferase; KR, Keto reductase and UGT, UDP-glucuronosyltransferase.

The Chilean soapbark tree (*Q. saponaria*) has been known to produce saponins, collectively known as QS saponins. These saponins in a mixture including QS-21, QS-17, and QS-7 are used as combination adjuvant for the COVID-19 vaccine produced by Novavax (NVX-CoV2373) [68]. QS-7 and QS-21 have the same biosynthetic core but vary in the modifications at the C-28 position. QS-7 contains a simple acetyl group and QS-21 has a long C18 acyl chain present at the C-28 position. Apart from this, the sugar moieties at the C-28 position are also different. QS-17 present in combination adjuvant is the glycosylated derivative of QS-21. Due to the structural complexity of these molecules, the only commercial resource is limited to the bark of the soapbark tree. However, the understanding of integrated approaches led to the complete elucidation of QS-7 and QS-21 from the soap bark tree [36, 37] (Figs. 3 and 4). Genome mining and combinatorial biosynthesis expression led to the identification of 16 pathway enzymes to make QS-7 in *N*. *benthamiana* [36]. The core structure for the biosynthesis of QS saponins is quillaic acid (QA), derived from the β-amyrin scaffold. Therefore, the pathway elucidation of QS saponins initiates by identifying β-amyrin synthase (QsbAS1). Further, transient expression of CYP716s (CYP716A224 and CYP716A297) along with QsbAS1 resulted in the formation of echinocystic acid. The gene CYP716A224 is characterized as C-28 oxidase and CYP716A297 as C-16α hydroxylase. The screening of P450s in the *Q. saponaria* transcriptome resulted in the identification of CYP714E52 as C-23 oxidase. The combinatorial expression of QSbAS1, CYP716A224, CYP716A297, and CYP714E52 resulted in the production of QA (Fig. 3). Furthermore, the biosynthetic chassis was utilized for the identification and characterization of a CSLM1 (cellulose synthase-like), an acyltransferase (QsACT1) and ~10 sugar transferases (UGT73CU3, UGT73CX1, UGT73CX2, UGT74BX1, UGT91AR1, UGT91AQ1, UGT73CY2, UGT73CY3, UGT73B44, and UGT91AP1), along with a fucose synthase (QsFucSyn) to form QS-7 in *N*. *benthamiana*. Indeed, QS-21 has a total of seven different glycosidic moieties that make the structure complex. Still, researchers have reconstructed the 20-step pathway in tobacco plants using combinatorial biosynthesis [37]. The QA-TriX-FRXX biosynthetic chassis genes were co-expressed with two ketoreductases (KR1 and KR2), two acyltransferases (ACT2 and ACT3), and one sugar transferase (UGT73CZ2) to form QS-21 (Fig. 4). Further, these enzymes were utilized to reconstruct the complete biosynthesis of QS-21 and its derivatives in engineered yeast strains [38]. These advancements in the biosynthesis of QS saponins open up an unprecedented path for the bioengineering of vaccine adjuvants. Further, the detailed understanding and investigation of these adjuvants promote the human immune response.

#### Boswellia serrata

**Figure 5:**
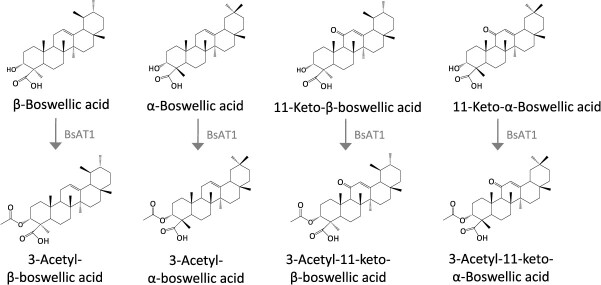
**Characterized acetyltransferase of *B. serrata* triterpene pathway.** Genes from *B. serrata* are shown in gray.

*Boswellia serrata* exudes the oleo-gum resins on wounding, which accumulates a vast range of triterpenes known as boswellic acids (BAs). The boswellic acids including αBA, βBA, 11-keto-βBA, 3-acetyl-αBA, 3-acetyl-βBA, and 3-acetyl-11-keto-βBA comprise a unique C3α-epimeric pentacyclic structure with medicinal properties. Recently, a wound-responsive BAHD acetyltransferase (BsAT1) was identified by conducting tissue-specific transcriptome profiling and gene function analysis [31]. BsAT1 is responsible for converting the αBA, βBA, and 11-keto-βBA to 3-acetyl-αBA, 3-acetyl-βBA, and 3-acetyl-11-keto-βBA in both *in vitro* and *in vivo* assays through C3α-O-acetylation reaction (Fig. 5). *BsAT1* transcript expression is also correlated with the enhanced level of C3α-O-acetyl-BAs in the bark in response to wounding. Although the initial step of epi-amyrin biosynthesis is still unknown, this study provides new insight into the biosynthesis of oleo-gum resin triterpene biosynthesis.

### Limonoids (*Azadirachta indica*, *Citrus sinensis*, and *Melia azedarach*)

Triterpenes are a diverse family of complex structural scaffolds with many pharmacological activities, including limonoids and quassinoids. Limonoids, also referred to as tetranortriterpenoids, are confined mainly to Spinadales plants, bolstering a notable structural diversity bearing a signature furan, with >2800 known structures [69]. The chemical synthesis of limonoids is not practical due to the different number of stereocenters and quaternary centers. However, few synthetic limonoid biosynthetic routes have been reported [70, 71], but the complete biosynthetic pathway to limonoids remained elusive. Recent efforts have been made to discover a photochemical approach for creating complex limonoid scaffolds through late-stage skeletal reorganization [72]. The lack of production limits the functional utility of these compounds as clinical candidates.

**Figure 6:**
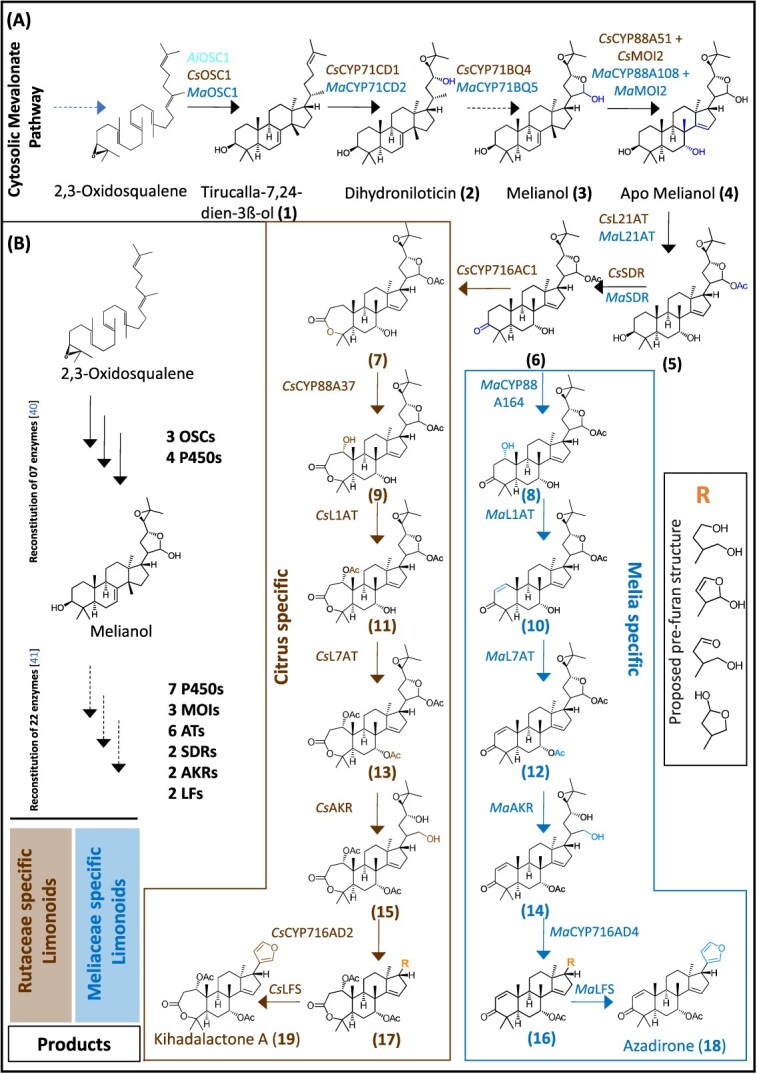
**Reconstitution of Rutaceae and Meliaceae limonoid biosynthetic pathway.** (A) Combinatorial expression of gene sets for the production of kihadalactone A [19] and azadirone [18] in *N*. *benthamiana* leaves. (B) Summarization of limonoid biosynthetic pathway. Genes from Citrus are shown in brown and those from Melia are shown in dark blue. Solid and dashed arrows denote single and multiple enzymatic steps, respectively. Abbreviations: OSC, Oxidosqualene cyclase; P450, cytochrome P450 monooxygenase; AT, acyltransferase; SDR, short chain dehydrogenase reductase; AKR, aldo-keto reductase; MOI, melianol oxide isomerase and LFS, limonoid furan synthase.

Recent studies utilize multi-omic approaches for identifying pathway genes of limonoid biosynthesis in *A. indica*, *C. sinensis*, and *M. azedarach* of Rutaceae and Meliaceae families [40, 41]. The genome (*C. sinensis* var. Valencia) and transcriptomes (*A. indica* and *M. azedarach*) were mined to elucidate the early steps of limonoid biosynthesis [40] (Fig. 6). The dataset mining results in the identification of an OSC that produces the 30-C precursor tirucalla-7,24-dien-3β-ol (**1**) from each of the three species (*Ai*OSC1/*Cs*OSC1/*Ma*OSC1). Furthermore, P450s from *C. sinensis* (CsCYP71CD1 and CsCYP71BQ4) and *M. azedarach* (MaCYP71CD2 and MaCYP71BQ5) were identified that are capable of oxidation of tirucalla-7,24-dien-3β-ol to form protolimonoid melianol (**3**) (Fig. 6A). This work acts as a base platform for identifying a biosynthetic route for protolimonoids and supports the pathway conservation between rutaceae and meliaceae families.

The generated platform is further utilized for the reconstruction of kihadalactone A and azadirone in *N*. *benthamiana* [41]. Additionally, publicly available microarray data for Citrus (Rutaceae) and extra RNA sequencing/ reference-quality genome (Meliaceae) were also used to discover biosynthetic routes to kihadalactone A and azadirone. In the case of *Citrus*, the *CsOSC1* gene is used as a bait gene for co-expression analysis on the *Citrus* fruit database. After the characterization of more biosynthetic genes, the list of bait genes is updated to identify other novel candidate genes. Similarly, *MaOSC1*, *MaCYP71CD2*, and *MaCYP71BQ5* were used as bait genes in sequenced tissues of Melia. The combined analysis leads to the selection of 17 candidate genes for the limonoid pathway (Fig. 6B). The co-expression of *CsCYP88A51* or *MaCYP88A108* with already characterized melianol biosynthetic genes led to the accumulation of multiple mono-oxidized products, suggesting that the resulting product is unstable or undergoes further modification. Further analysis resulted in the identification of four sterol isomerases (named melianol oxide isomerases, MOIs), CsMOI1 to CsMOI3 and MaMOI2 that produce mono-oxidized melianol isomer products. Rigorous co-expression analysis on CsMOIs/MaMOIs resulted in the identification of CsMOI2, CsMOI3, and MaMOI2, responsible for forming C-14/15 double-bond classic limonoid scaffold, while CsMOI1 forms cyclopropane ring scaffold for glabretal limonoids. The reconstruction experiment concludes that CsCYP88A51, MaCYP88A108, and two different types of MOIs are responsible for converting melianol (**3**) to apo-melianol (**4**) via epoxide intermediate in the classical limonoid pathway.

After the identification of enzymes involved in methyl shift in limonoids, the enzymes for acetylation and dehydrogenation of apo-melianol were screened. The screening analysis identified BAHD-type limonoid 21-O-acetyltransferase (CsL21AT or MaL21AT) and short-chain dehydrogenase reductases (CsSDR or MaSDR). The coexpression of genes in *N*. *benthamiana* results in the loss of compound apo-melianol to acetylated (**5**) and dehydrogenated (**6**) products. Further metabolite analysis showed that L21AT could utilize compounds 1 and 3, and SDR is active on all intermediates after the OSC1 products. The subsequent screening process revealed further activity of P450s. Two P450s, CsCYP716AC1 and CsCYP88A37 from *Citrus,* were identified that catalyzed **6** to produce **7** and **8** or constitutively to **9** and one P450 from Melia, *Ma*CYP88A164, which is also able to oxidize **6** to **8.** Further steps in both *Melia* and *Citrus* candidate gene screens revealed BAHD acetyltransferase activity. *Cs*L1AT and MaL1AT acetylate C-1 hydroxyls of **9** and **8** to form **11** and **10**. Two more AT homologs, *Cs*L7AT and *Ma*L7AT (named limonoid 7-O-AT), were active on **11** and **10** to generate acetylated scaffolds **13** and **12.**

Further screen analysis for enzymes involved in the downstream pathway, which involves C-4 scission implicated in a furan ring formation, remains elusive. The screening of candidate genes led to the identification of three active candidate pairs, the aldo-keto reductases (AKRs) (CsAKR/MaAKR), the CYP716ADs (CsCYP716AD2/ MaCYP716AD4), and the 2-ODDs [named limonoid furan synthase (LFS), CsLFS/ MaLFS]. The systematic testing of these gene sets through agro-infiltration resulted in the accumulation of the furan-containing molecules, kihadalactone A (**19**) and azadirone (**18**) (Fig. 6A), which are present in respective native species. Overall, the enzymatic platform is utilized for the discovery of 22 enzymes to reconstruct kihadalactone A and azadirone pathway in *N*. *benthamiana* (Fig. 6B). The cumulative results enable access to limonoid biosynthesis and provide a template for complex triterpene pathway discovery and reconstitution.

Refactoring complex triterpene biosynthetic pathways in heterologous hosts could promise more access to clinically important candidates such as quassinoids, quinonoids, and Schisandra nortriterpenes, and new-to-nature derivatives. The biosynthetic routes for these compounds are yet to be determined. These compounds are either restricted to a single species or produced through multiple complex biosynthetic steps, which limit the drug development process. The method used in the study demonstrates the refactoring of a very complex pathway into *N*. *benthamiana* for rapid production and isolation of limonoids. Furthermore, this synthetic biology platform would be used as an attractive method for generating complex limonoid scaffolds. The stable bioengineering process could be utilized as a viable strategy for sustainable crop protection.

### Design-build-test-learn (DBTL) platform for triterpene bioengineering

**Figure 7:**
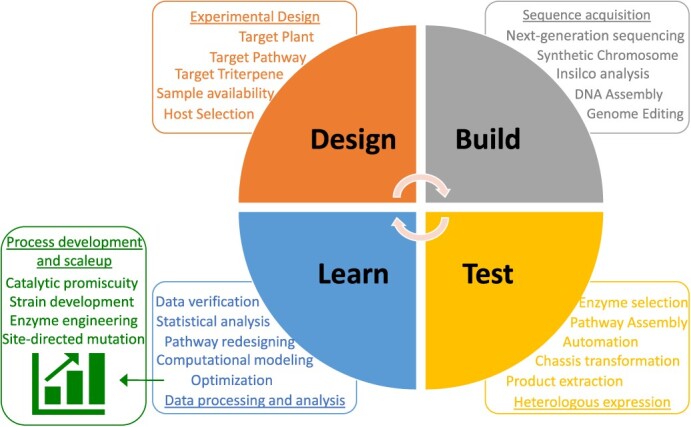
**Schematic of Design-Build-Test-Learn (DBTL) cycle in triterpene biosynthetic pathway**. The DBTL cycle is a platform in synthetic biology utilized for developing and testing heterologous hosts with desired functionalities. Over the years, bottlenecks in different steps of the DBTL cycle of triterpene biosynthesis have been resolved, enabling advancements in development and scaleup processes.

The continuous research on elucidating the natural product biosynthesis pathways has rendered the production of rare triterpene using synthetic biology platforms. These platforms utilize heterologous hosts for the biomanufacturing of next-generation chemical production [73, 74]. By 2030, the biomanufacturing industry could reach $30 trillion in the global economy [75]. The microbial biomanufacturing of commodity chemicals promises to reduce greenhouse emissions and enhance sustainability by providing alternatives from renewable feedstocks [76]. However, the limited industrial implementation is attributed toward the slow production rates and time-intensive methods of strain engineering. Enabling the Design-Build-Test-Learn (DBTL) technologies in synthetic biology is useful for achieving rapid and selective bioengineering to produce biopharmaceuticals, that meet human needs. With the exponential increase in DNA sequence information and computational tools, many putative gene clusters have been identified. However, to date, only a few of them have been discovered for natural products. The major bottlenecks are the low expression of these clusters and the disruption of intracellular homeostasis in heterologous hosts under laboratory conditions. In the past few decades, the demand for bio-based products has increased and synthetic biology helps in delivering the products quickly. The multi-omics and synthetic biology approach expedites bioproduction development by identifying target compounds, genes/enzymes, integration into heterologous hosts, strains engineering, and model optimization (Fig. 7 and Table 2). DNA synthesis, genome/transcriptome sequencing, genome editing, and metabolic engineering tools have greatly accelerated the DBTL phase processes (Table 1). Even the integration of artificial intelligence and machine learning in combination with metabolomics is routinely performed during the production of target compounds [74,77,78]. Nowadays, deep-learning user-friendly toolkits such as BioNavi-NP are available that help to predict natural products and natural products like biosynthetic pathways [79]. BioNavi-NP uses a single-step bio-retrosynthesis prediction model to identify plausible biosynthetic pathways through an AND-OR tree-based planning algorithm. Thorough evaluation reveals that the toolkit can identify 90.2% of 368 test compounds with a recovery of reported building blocks 1.7 times more accurately than conventional-rule-based approaches. Due to low abundance in their native plant as a complex mixture of structural/stereo-isomers and limited feasibility of chemical synthesis, bioengineering in heterologous hosts following biosynthetic pathway modulation is the most promising strategy. The DBTL cycles serve as a standard workflow for creating and scaling up the process for industrial production of triterpene compounds. Still, these systems face specific challenges such as scalability issues, unsuitable microenvironment, feedback inhibition, and species/tissue/developmental stage-specific biosynthetic peculiarities. Therefore, several technological advancements are needed in each component of DBTLc to work in a better way. To date, minor progress has been made toward implementing DBTL pipelines for tree species and this needs to be discovered in future. Here, the steps involved in the DBTL cycle are discussed in the context of triterpene biosynthesis. Several examples in which strains are modified to produce the desired triterpenes in high titer are also discussed.

**Table 2:** DBTL strategies utilized for bioproduction of triterpenes.

**Strategies used**	**Triterpene**	**Yield**	**Heterologous hosts**	**References**
Site-directed mutagenesis and expression of squalene synthase	Ambrein	2.6 mg/l	*E. coli*	[80]
Reconstruction of biosynthetic pathway	Dammarenediol-II	8.63 mg/l	*E. coli*	[81]
Extension of cell membrane by overexpression of membrane proteins	Squalene	612 mg/l	*E. coli*	[82]
Reconstruction of biosynthesis pathway and glycerol catalytic pathway	Ginsenoside CK	433.1 mg/l	*S. cerevisiae*	[83]
Reconstruction of PPD production chassis, optimization of UGTPg1 expression, and tuning down UDP-glucose consumption	Ginsenoside CK	5.74 g/l (fed-batch fermentation)	*S. cerevisiae*	[84]
P450-CPR enzyme fusion and MVA pathway gene overexpression	Oleanolic acid	540.7 mg/l	*Y*. *lipolytica*	[85]
Modular engineering of MVA pathway and optimization of P450 expression levels	PPD	529 mg/l (shake flasks) and 11.02 g/l (fed-batch fermentation)	*S. cerevisiae*	[86]
Remodeling of homologous recombination efficiency and integration of MVA pathway into the chromosome	Squalene	35 g/l	*Y*. *lipolytica*	[87]
Manipulation of lipid components	Lupeol	411.72 mg/l (shake flasks)	*Y*. *lipolytica*	[88]
Integration of endogenous pathway genes and knockout of inhibiting genes by CRISPR/Cas9 technology	Friedelin	63.91 mg/l (shake flasks)	*S. cerevisiae*	[89]
Reconstruction of the triterpene pathway	Ginsenoside Rh2	354.69 mg/l (shake flasks)	*S. cerevisiae*	[90]
Mitigation of the inhibitory effect of squalene and peroxisome compartmentalization	β-amyrin	347 mg/l (shake flasks) and 2.6 g/l (fed-batch fermentation)	*S. cerevisiae*	[91]
Reconstitution of triterpene pathway	Betulinic acid, corosolic acid, and maslinic acid	3.09 μg/g FW, 11.94 μg/g FW, and 4.38 μg/g FW	*N*. *benthamiana*	[33]
Co-expression of triterpene pathway genes	12,13β-epoxy-16β-hydroxy-β-amyrin	1.18 mg/g DW	*N*. *benthamiana*	[92]
Multigene stacking through in-fusion-based gene stacking strategy (IGS) for the generation of transgenic plants	Mogroside III and mogroside II-E	148.30–252.73 ng/g FW and 339.27–5663.55 ng/g FW	*N*. *benthamiana*	[93]
	Siamenoside I and mogroside III	29.65–1036.96 ng/g FW and 202.75 ng/g FW	*A. thaliana*	[93]

### Design

The DBTL workflow begins with the experimental design including identification of target plant, target compound, target pathway, and host selection, in which an appropriate host is selected based on past knowledge and metabolic maps. Further, the potential enzymatic modifications in the target pathways are proposed. Advances in synthetic biology and technological automation facilitate the production of natural products in various DBTL phases. In the case of triterpene, both prokaryotes and eukaryotes can be used as heterologous hosts [1, 2, 6]. In bacteria, squalene directly acts as a triterpene precursor to form hopane-type triterpenoids [8]. However, in eukaryotes, the 2,3-oxidosqualene and analog (3S,22S)-2,3:22,23-dioxidosqualene have been cyclized to form diverse triterpene structures [94]. Furthermore, the combinatorial expression of biosynthetic enzymes and bioengineering opens the prospects for harnessing triterpene in high titers. The major challenge in the reconstitution of the complex triterpene pathways is to determine whether the enzymatic transformation is active ‘on targeted pathway’ or heterologous hosts. To check these, the bait genes were used to enhance the co-expression analysis stringency and refine the candidate list. Further, the top-ranking genes based on Pearson’s correlation coefficient to the bait genes were tested for their functional role through coexpression in *S. cerevisiae* or agro-infiltration in *N*. *benthamiana* [41].

### Build

Next, the database is built to get information on the experimental design. These include building the data resources such as elucidation strategies, strain construction, and method development, leading to pathway design. The presence of complex genomes in plants makes it hard to identify the genes involved in the target triterpene biosynthetic pathway. The complexity is further increased in finding the right enzymes for specific biosynthetic pathways and reactions. All these complications make the screening process more laborious and time-consuming. Traditional gene discovery methods such as gene deletion, gene silencing, and *in vitro* enzymatic assay have various shortcomings. Nowadays, various integrated OMICS approaches such as genomics, transcriptomics, and proteomics generate huge datasets and accelerate the pathway discovery process (Table 1). The triterpene biosynthetic genes are scattered along plant chromosomes (*Barbarea vulgaris*) making the process of pathway elucidation more challenging. However, in several cases, the triterpene biosynthetic genes are also organized in gene clusters (*Arabidopsis thaliana*, *Avena strigosa*, *Cucurbitaceae*, *Lotus japonicus*, and *Solanum spp*.), making the process of gene discovery faster. Further, the data is analyzed through computational tools to annotate it with available databases, including uniport, KEGG, P450s, and UGTs for the identification of target enzyme classes. Strain and vector are also constructed in the build phase of the DBTL platform. Collectively, these methods along with coexpression data and sequence-based prediction methods provide a new dimension to gene discovery techniques.

### Test

Subsequently, the constructed strains and predicted enzymes are evaluated during the test phase. Particularly, in the test phase all genes/enzymes are rewired to form desired triterpenes. The test phase includes various strategies such as manipulation of host metabolism, bioengineering of target enzymes, and subcellular compartmentalization at the organelle level. The products produced were checked with advanced analytical tools such as GC–MS (Gas Chromatography–Mass Spectrometry), HPLC (High-performance liquid chromatography), NMR (Nuclear magnetic resonance), etc, and reports were analyzed and verified. Successful implementation of DBTL phases produces triterpenes in a larger amount and acts as an alternative method. For instance, the synthetic biology-based engineering of *S. cerevisiae*, through reconstruction of ginsenoside compound K (CK) biosynthesis and glycerol catabolic pathway, resulted in the increased production of CK to 433.1 ± 8.3 mg/l. The transcriptome analysis of the engineered strain revealed the genes involved in the MVA and the uridine diphosphate glucose (UDPG) synthesis were upregulated, resulting in 2.4 times product formation in comparison with glucose medium [83]. Similarly, heterologous hosts were used to reconstruct the biosynthetic pathway by expressing OSCs, P450s, UGTs, and ATs for QS saponins, cucurbitacins, ginsenosides, mogrosides, limonoids, betulinic acid, oleanolic acid, maslinic acid, ursolic acid, corosolic acid, type II ganoderic acids, and morolic acid [ 1, 33, 34, 36–38, 40, 41, 95–98].

As a green factory, the native plant can produce natural products at a very low cost by utilizing light, water, air, and soil. Traditional bioengineering approaches focus on pathway optimization, yet are often complicated by downstream consumption and product toxicity. The presence of different cellular organelles further benefits metabolic engineers and synthetic biologists, as they can provide diverse microenvironments suitable as per the enzyme requirement [99]. Further, utilization and modification of subcellular compartments to produce triterpenes by synthetic biology have good application prospects. For instance, yeast PLN1 protein targets protopanaxadiol (PPD) synthase to lipid droplets, resulting in increased efficiency by 394%. Furthermore, the production chassis is utilized to produce the PPD-type saponin ginsenoside compound K (CK) to reach 5 g/l in 5-l fed-batch fermentor [100]. Similarly, squalene titer is increased to 1312.82 mg/l through peroxisome engineering, which is 138-fold higher than the native yeast strain [101]. Additionally, modulation through cytoplasm engineering and optimization of a two-stage fed-batch fermentation produce squalene to 11.00 g/l. Similarly, reconstruction of the squalene biosynthetic pathway in peroxisome led to increasing the squalene level to 1300-fold in *Yarrowia lipolytica* [102]. In bioreactor fermentation, these strategies produce a squalene yield of 32.8 g/l from glucose and 31.6 g/l from acetate. Even these micro-compartments were targeted to produce triterpenes in high titer such as squalene and botryococcene in transgenic plants [103, 104]. The plastidial squalene biosynthesis is also targeted in transgenic poplar to produce 0.63 mg/g fresh weight in leaf tissue [105]. Nowadays, CRISPR/Cas9 is a commonly used technique for genome modification in homologous and heterologous hosts for plant-derived triterpene production. For example, knock out of inhibiting genes (*BTS1*, *ROX1*, *YPL062w*, and *YJL064w*) through CRISPR/Cas9 technology along with the integration of endogenous pathway genes (*tHMG1*, *ERG1*, *ERG20*, *ERG9*, *POS5*, or *UPC2.1*), resulted in 63.91 ± 2.45 mg/l (65-fold) friedelin production in yeast [89]. The double knockout of CYP93E3 and CYP72A566 through genome editing along with CYP88D6-overexpression leads to a 3-fold increase (∼1.4 mg/g) in glycyrrhizin accumulation in hairy roots [106]. Further, this technology is useful for the identification of the biological functions of triterpene biosynthetic genes. For instance, the deletion of the squalene epoxidase gene (PcSE) using CRISPR/Cas9 results in the inhibition of triterpene biosynthesis in *Poria cocos* protoplasts [107]. Similarly, the deletion of GuCSyGT in licorice hairy roots using CRISPR-Cas9-mediated genome editing results in the complete absence of soyasaponin I, demonstrating its role in saponin biosynthesis [108]. Overall, these strategies lead to the production of several triterpenes in high titer (Table 2), and approaches such as site-directed mutagenesis and rational designing would be implemented for altered triterpene content.

### Learn

By analysis of the test results, metabolite rules are extracted, providing valuable feedback for the next design iteration. A relation is established between pathway design and production in the learning phase. For example, a recent study found that altering the yeast fermentation environment to 22°C resulted in increased production and efflux of glycyrrhetinic acid (GA), β-caryophyllene, and α-amyrin [109]. Production of GA is enhanced by 5.5-fold compared with yeast growing at 30°C. This study is interesting as a small temperature change resulted in the optimization of the whole fermentation process and increased production. Similarly, light and low temperatures induce the production of carotenoids in *Neurospora crassa* at 8°C, resulting in a 15-fold increase in content compared to the conventional methods [110]. Therefore, it is suggested that employing environmental variables to produce natural products can be a flexible approach, compared to complex genetic modification. Further, this data is used to inform the next DBTL cycle and enhance the production per rotation cycle.

## Conclusion and future perspective

Triterpene represents a vast and structurally diverse class with >100 cyclic scaffolds and >20 000 structures. The triterpene scaffolds are produced from 2,3-oxidosqualene through OSC-mediated cyclization reactions. Further triterpene diversity is multiplied by modifications incorporated with the other groups of enzymes such as P450s, UGTs, and ATs. Considering the diversity of triterpene structures, it is quite evident that the enzymes involved in biosynthesis are yet to be identified. Recent technologies such as integrated OMICS, advanced computational biology, and chemical analytics have greatly increased the valuable data from non-model plants, resulting in increased counts in triterpene biosynthetic enzymes. From the identified triterpene biosynthetic enzymes, the majority are characterized from herbaceous plants. Some triterpenes such as ursonic acid and arjunolic acid are produced in horticultural trees including ornamental, landscape, fruit, and nut production, and have good bioactivities, but their biosynthetic pathways have not yet been elucidated. Therefore, it is still reasonable that the tally of triterpene biosynthetic enzymes will increase for horticultural trees in the future. The availability of huge genome and transcriptomic data facilitates system biologists and computational biologists to accelerate the gene discovery processes, further improving the chances of multidisciplinary collaborations. The increasing demand for these highly valuable triterpenoids has led to the growing interest of synthetic biologists across the globe to produce them in ‘free-from-tree’ strategies. Reconstruction of the triterpene pathway by combinatorial biosynthesis and engineering in heterologous hosts offered an alternative strategy to achieve increased production and new-to-nature compounds. Further, the pattern identification and introduction of a DBTL framework toward biotechnological production are expected to help in targeting specific triterpenes with the desired yield. At present, synthetic biologists mainly utilize heterologous hosts that are capable of reconstructing the triterpene pathways in a step-by-step co-expression manner and help in targeting different combinations of enzymes. Additionally, these are easily tractable and metabolically less complex than native plants; therefore, might be scalable for triterpene production. DBTL platform helps in producing the triterpenoids in high titer through different combination strategies, including the revamping of triterpene precursor/cofactor supply, blocking the precursor used in competitive pathways and harnessing cellular compartments along with combinatorial expression of biosynthetic genes and regulators. Moreover, the identification of new heterologous hosts such as photosynthetic microorganisms (e.g. algae and cyanobacteria) utilizing renewable energy and carbon sources could be a better alternative in the future.

Although the integrated approaches led to substantial progress toward the elucidation of triterpene biosynthesis, the biological functions are still not understood. The implementation of tools such as CRISPR/Cas9 and gene overexpression in non-model plants is important for understanding the biological roles. It is expected that many unknown multidisciplinary fields are yet to be explored for triterpene studies in horticultural trees. This review aims to lay a foundation for identifying triterpene biosynthetic pathways and offers new capabilities for sustainable production in heterologous hosts. The future challenge is to identify novel uncharacterized triterpene biosynthetic pathways and generate an optimized DBTL framework for sustainable triterpene production. Further, research on complex triterpene pathways and specific regulators/transporters can break these bottlenecks and provide a sustainable production system.

## Data Availability

The author confirms that all data in this study are available and can be found in this article.
